# 

**Published:** 1884-06

**Authors:** 


### CONTENTS:

ORIGINAL COMMUNICATIONS:

PAGE.

I. Doctorate Address Delivered at the Commencement of the Woman's Medical College, by Wm. H. Byford, A.M., M.D., President, on April 22, 1884 ......... 561

II. Paretic Dementia and Life Insurance. By James G. Kiernan, M.D., Chicago .... 574

III. Clinical and Pathological Reports of Cases of Insanity. By S. V. Clevenger, M. D ........ 583

IV. The Removal of Naso-Pharyngeal Fibromata by the Galvano-Cautery, or Steel Wire Écraseur. By E. Fletcher Ingals, A.M.M.D., Chicago ........ 604

PAGE.

V. On Gelsemium Sempervirens. By Charles Gilbert Davis, M.D., of Chicago ........ 610

SOCIETY REPORTS:

At the Chicago Medical Society ........ 631

Chicago Medical Society ........ 643

Michigan State Board of Health ........ 655

EDITORIAL:

Dr. Dearborn ........ 658

DOMESTIC CORRESPONDENCE ........ 661

REVIEWS ........ 666

SELECTIONS:

Hospital with Circular Wards ........ 671

OBITUARY ........ 657

ERRATA ........ 660

ITEM ........ 630

